# Editorial: Impact of System Biology and Molecular Medicine on the Management of Complex Immune Mediated Respiratory Diseases

**DOI:** 10.3389/fmed.2021.745739

**Published:** 2021-09-08

**Authors:** Blanca Cárdaba, Girolamo Pelaia

**Affiliations:** ^1^Immunology Department, Instituto de Investigación Sanitaria-Fundación Jiménez Díaz, Universidad Autónoma de Madrid (UAM), Madrid, Spain; ^2^Center for Biomedical Network of Respiratory Diseases (CIBERES), Instituto de Salud Carlos III, Madrid, Spain; ^3^Department of Health Sciences, University “Magna Græcia” of Catanzaro, Catanzaro, Italy

**Keywords:** asthma, chronic obstructive pulmonary disease, COPD, respiratory inflammation, systems biology, biological therapies

Chronic respiratory disorders, including bronchial asthma and chronic obstructive pulmonary disease (COPD), are common, highly complex and heterogeneous inflammatory diseases that include a broad clinical spectrum. These disorders could be caused by numerous genetic, pharmacologic, physiologic, biological, and/or immunologic mechanisms, giving rise to subclasses of phenotypes and endotypes. This great heterogeneity is reflected in the absence of a good therapeutic option for a substantial percentage of patients and is behind extensive studies aimed at applying precision or personalized medicine, which requires different diagnostic and therapeutic approaches, in the field. In this Research Topic we aim to provide an overview of the latest advances in the search for new approaches to discover and validate new tools related with diagnosis, prognosis, exacerbations, and treatment monitoring as well as molecular targets for new biological therapies.

Twelve articles have been published, summarizing different aspects related mainly with asthma and COPD. Seven report the results of human studies and five are examples of the relevance of animal models in the knowledge base on respiratory disorders.

Starting with studies in humans, Zhang et al. prove the efficacy of single allergen (*Dermatophagoides farinae*) sublingual immunotherapy (SLIT) in polysensitized patients allergic to *D. farinae* with allergic asthma. They describe a 3-year longitudinal case-control study with three kinds of cases: the single-allergen group [only sensitized to house dust mite (HDM)], the 1–2 allergen group (HDM combined with 1–2 other allergens), and the 3 or more allergens group (HDM combined with 3 or more other allergens). Their results show an improvement in patients sensitized to HDM with and without other allergens after 3 years of SLIT, but with a slower recovery of symptoms in patients with 3 or more allergen sensitizations.

Pelaia et al. provide an exhaustive overview of asthma complexity, focusing on new molecular therapeutics options according to the pathobiological mechanisms underlying the various cellular and molecular phenotypes of severe asthma. They underscore the relevant improvement in the management of patients with severe, allergic, or non-allergic eosinophilic T2-high asthma, especially in that their susceptibility to suffer from frequent and often serious disease exacerbations is lessened due to the new drugs approved, anti-IgE, anti-IL-5, anti-IL-5 receptor, and anti-IL-4/IL-13, all receptor monoclonal antibodies, and the promising new monoclonal antibodies, mainly targeting the innate cytokines known as alarmins, which are in development stage. However, they highlight the need to increase the search for new molecular targets for patients with severe T2-low asthma (neutrophilic or paucigranulocytic asthma) who currently are largely excluded from the therapeutic benefits achievable for people who experience T2-high severe disease.

Ragnoli et al. focus on other prevalent respiratory diseases, obstructive sleep apnea syndrome (OSAS), which is currently underdiagnosed. They review the model that proposes a bidirectional correlation between severe asthma (SA) and OSAS, with a mutual negative effect in terms of disease severity. These two diseases share common risk factors, such as obesity, rhinitis, and gastroesophageal reflux (GER). Also, it has been proposed that OSAS and asthma patients may be more susceptible to SA attacks induced by systemic inflammation. In their review, the authors describe the published evidence on the interrelationship between OSAS and SA, from endo-phenotype to clinical aspects, highlighting possible implications for clinical practice and future research directions, remarking that OSAS treatment can also improve lung function tests in adult asthmatic patients and that assessing the coexistence of OSAS and SA could help in the management of both diseases.

The next 4 articles (Cremades-Jimeno et al.; Kim et al.; Ricciardi et al.; Cañas et al.) are examples of how the new bioinformatic tools and systems biology approaches are helping to describe new molecular biomarkers or molecular motifs of diseases and new gene regulatory networks or specific regulatory elements (RNA-binding proteins or RBPs, Micro-RNAS, or miRNAs), which are opening up new fields to better understand and manage these complex diseases.

Cremades-Jimeno et al. used systems biology approaches to define molecular motifs of three diseases, allergic asthma (AA), non-allergic asthma (NA), and respiratory allergy (RA) and categorize the relevance of molecular biomarkers previously defined experimentally in peripheral blood mononuclear cells (PBMCs) in order to prioritize them according to their relationship to specific molecular motifs or to disease. The study provides new information on potential biomarkers with a mechanistic implication, opening a new focus with which to find diagnostic and therapeutic tools for these types of diseases.

Kim et al. assess gene regulatory networks (how the transcription factors (TFs) regulate gene expression to determine the responsiveness to anti-asthma therapy) of adult patients with asthma who showed good or poor lung function improvements in response to inhaled corticosteroids (ICSs). Their results indicated that responses between good and poor responders to a certain drug is not necessarily derived from differential gene expression networks but may instead be from regulatory gene expression networks. They identified TFs that showed different gene connections and enrichment in distinct biological pathways, remarking TGF-β signaling, cell cycle related, and IL-4 and IL-13 signaling pathways that could be important in determining responses to ICSs in patients with asthma.

Regarding severe asthma and COPD, Ricciardi et al. describe an interesting *in silico* analysis of posttranscriptional gene regulation using a curated list of mRNA-binding RNA Binding Proteins (mRBPs) in selected Gene Expression Omnibus (GEO) transcriptomic databases of airway epithelium isolated from chronic obstructive pulmonary disease (COPD), severe asthma (SA), and matched control subjects. This study identifies an overall downregulation of RBP expression that was shared by a subset of smoker control subjects; changes in mRBP expression impacted several biological pathways also involved in several aspects of COPD pathogenesis; finally, at least five groups of coregulated RBPs were identified. Importantly, airway epithelial mRBP expression was found to be much less regulated in patients with SA. Overall, the COPD-related mRBP profile found in this study suggests post-transcriptional control of epithelial gene expression as substantial, yet understudied process possibly contributing to key pathogenic mechanisms in COPD.

RNA-binding proteins exert their function as part of ribonucleoprotein (mRNP) complexes, constituted by proteins and non-coding RNAs such as microRNAs (miRNAs). In this Research Topic, Cañas et al. contribute an elegant review of the current knowledge and promising roles of miRNAs (small non-coding RNAs) in pathophysiology, diagnosis, and treatment of asthma and COPD. This paper exhaustively surveys recent descriptions of differential expression of miRNAs in peripheral and target-derived samples of asthma and COPD patients focusing on the growth potential of these regulatory elements in relation to key aspects of those diseases and how their complexity could be better understood with a systems biology approach to compile and elucidate the complex miRNA regulatory system to define their usefulness in the management of these diseases.

Finally, five articles describe relevant aspects of respiratory disorders by experimental animal models.

Foray et al. describe a new house dust mite-induced asthma model to elucidate allergic dysimmune mechanisms involving Th2 and Th17 responses that could better mimic some asthmatic endoytpes. In this study, non-obese diabetic (NOD) mice, which spontaneously develop autoimmune diabetes, undergo more severe allergic asthma airway inflammation and airway hyperresponsiveness (AHR) than pro-Th2 BALB/c mice upon house dust mite (HDM) sensitization and challenge. The use of IL-4-deficient NOD mice and the *in vivo* neutralization of IL-17 demonstrated that both IL-4 and IL-17 are responsible for the exacerbated airway inflammation and AHR observed in NOD mice. Although further studies are required to better determine whether Th2 and Th17 responses act together or independently to induce vast airway inflammation and AHR in NOD mice, this could represent a unique model to better understand the key mechanisms implicated in the association between allergic airway inflammation and autoimmune diabetes, as well as to understand several asthmatic phenotypes with mixed Th2 and Th17 inflammation.

Lertnimitphun et al. analyze the effectiveness of Safranal—one of the active compounds from *Crocus sativus*, known in Chinese herbal medicine to have many anti-inflammatory properties—in two allergic murine models: ovalbumin (OVA)-induced asthma and passive systemic anaphylaxis (PSA) models. This paper demonstrates that Safranal has potential to stabilize mast cells and inhibit cytokine production through the inhibition of the MAPKs and NF-κB pathways, being a potential candidate to treat allergic diseases such as systemic anaphylaxis and asthma.

Finally, three interesting articles study new therapies for acute lung injury (ALI)/acute respiratory distress syndrome (ARDS), a serious illness characterized by severe pulmonary edema and a profound inflammatory response in the lung. Life-threatening acute lung inflammation can be induced by diverse ischemia/reperfusion (IR) injury conditions, including resuscitation for cardiac arrest, cardiopulmonary bypass, hemorrhagic shock, pulmonary embolism, and lung transplantation. Here, three articles describe animal models to test new treatments using different ALI inductors.

Liao et al. using a perfused rat lung model (ischemia/reperfusion (IR)-induced acute lung inflammation), analyze the effect of a potent-anti-inflammatory agent, 2-Methoxyestradiol (2ME), a natural 17-β estradiol metabolite. Also, its correlation with Annexin A1, a glucocorticoid-regulated protein that reduces vascular inflammatory responses associated with IR injury, was analyzed. Their results show that 2ME ameliorates IR-induced acute lung inflammation by upregulating the expression of endogenous AnxA1 in the lungs, alveolar epithelial cells, and neutrophils.

Pao et al. analyzed the influence of endoplasmic reticulum (ER) stress inhibitor, 4-phenyl butyric acid (4-PBA), a chemical chaperone, in mice with hyperoxia-induced acute lung injury (HALI). Also, its correlation with claudin-4 protein (a member of integral membrane proteins that are essential components in the tight junction (TJ) formation and function) was analyzed. In this study, 4-PBA effectively reduced oxidative and ER stress, the level of proinflammatory cytokines, and apoptosis, but increased claudin 4 protein expression in HALI. 4-PBA significantly improved multiple indices of HALI, such as prolonging survival, and decreasing AFC, lung edema, and disruption of tight junction proteins, production of pro-inflammatory cytokines, oxidative stress, the pulmonary neutrophil influx, and lung tissue damage. Consistent with *in vivo* findings, 4-PBA treatment had a similar advantageous effect on *in vitro* epithelial cells exposed to hyperoxia, however, these protective effects of 4-PBA were abolished when claudin-4 was knocked down. These experiments indicate that 4-PBA may have potential benefits as adjuvant therapy for HALI and the protective mechanism was *via* enhancing claudin-4 expression.

Tai et al. analyzed a new treatment, tanshinone IIA (TIIA), the main active ingredient in *Salvia miltiorrhiza* Bge, combined with cyclosporine A (CsA) in a model of obese rats with induced renal ischemia-reperfusion (IR). The authors analyze lung apoptosis led by renal IR and evaluate whether this combination could alleviate lung apoptosis by regulating mitochondrial function through the PI3K/Akt/Bad pathway in obese rats. Their results demonstrate that lung mitochondrial dysfunction was induced in the process of renal IR, especially in obese rats, with dynamics altered and biogenesis inhibited. TIIA+CsA were protective agent, which can attenuate lung apoptosis via modulating mitochondrial function by activating the PI3K/Akt/Bad pathway in obese rats. These results may be a promising protective strategy for managing obesity-related acute kidney injury and acute lung injury. However, this application needs further large-scale experimental and clinical studies.

Globally, these 12 articles show a brushstroke on the multiple not yet well-defined aspects, related to the molecular bases underlying to these complex respiratory diseases, which need to be validated in more extensive clinical studies to be clinically translated in a near future. In summary, this Research Topic reflects the high complexity of respiratory diseases and the many efforts, from different points of view, that are needed to improve the management of these disorders, which affect a high number of subjects around the world. Thank you to the authors and researchers who have contributed to this topic and we hope that this issue helps readers understand the extent to which efforts in this field are needed to offer best quality of life to patients.

## Author Contributions

BC and GP wrote and reviewed the manuscript. Both authors contributed to the article and approved the submitted version.

## Funding

This work was supported by research grant PI20/00903 cofunded by FEDER, CIBERES (ISCIII, 0013), and RETIC (RD09/0076/00101) from the *Fondo de Investigación Sanitaria* (*Ministerio de Sanidad y Consumo*, Spain) and in part by the research grant *Ayudas de la Sociedad Española de Alergia e Inmunolog*í*a Cl*í*nica* (SEAIC 2018).

## Conflict of Interest

The authors declare that the research was conducted in the absence of any commercial or financial relationships that could be construed as a potential conflict of interest.

## Publisher's Note

All claims expressed in this article are solely those of the authors and do not necessarily represent those of their affiliated organizations, or those of the publisher, the editors and the reviewers. Any product that may be evaluated in this article, or claim that may be made by its manufacturer, is not guaranteed or endorsed by the publisher.

